# Sensitivity analysis and integrated optimization of placement and parameters of rotational friction dampers based on seismic energy approach considering soil-structure interaction

**DOI:** 10.1371/journal.pone.0331741

**Published:** 2025-10-30

**Authors:** Hossein Jarrahi, S. Javad Vaziri, Mohsen Khatibinia

**Affiliations:** 1 Department of Civil Engineering, University of Birjand, Birjand, Iran; 2 Department of Civil Engineering, Semnan University, Semnan, Iran; Universiti Teknologi Malaysia, MALAYSIA

## Abstract

The optimization of seismic resilience in steel moment-resisting frames (SMRFs) has driven extensive research into passive energy dissipation systems, particularly rotational friction dampers (RFDs). This study introduces a novel hybrid optimization framework that simultaneously determines the optimal placement and design parameters of RFDs in SMRFs. A sensitivity analysis of key RFD parameters, including frictional moment and rigid beam length, highlights their influence on seismic performance. The optimization problem is formulated based on the seismic energy dissipation concept, employing a modified binary and real-coded particle swarm optimization (BRPSO) algorithm. The study examines both 6- and 10-story SMRFs under artificial earthquake records, incorporating soil-structure interaction (SSI) effects to enhance accuracy. The results reveal that the optimal distribution of RFDs significantly reduces structural hysteretic energy and inter-story drift while improving energy dissipation efficiency. A comparative analysis shows that, for both SMRF configurations, an increase in the number of RFDs beyond the optimal threshold yields diminishing benefits in seismic mitigation. The proposed framework not only enhances structural resilience but also minimizes construction costs by ensuring an efficient damper layout. These findings provide valuable insights for designing cost-effective and high-performance seismic protection strategies for mid- to high-rise structures.

## 1. Introduction

Recent advances in earthquake engineering focus not only on providing sufficient strength to structures but also on minimizing damage when they are subjected to seismic events [1–4]. The limited capacity of conventional seismic design codes to effectively dissipate seismic energy has driven the advancement and adoption of passive control devices for both upgrading existing structures and designing new ones, aiming to enhance their seismic resilience, safety, and performance [5–9].

Tuned mass dampers (TMDs) are among the most extensively employed seismic energy dissipation devices utilized in the structural engineering domain to alleviate earthquake-induced forces in buildings and infrastructure. These devices, which function based on the principle of transferring vibrational energy to an auxiliary mass system, have long been recognized as effective passive control mechanisms for attenuating undesirable structural vibrations. Numerous studies have underscored the efficacy of TMDs in enhancing the dynamic performance of structures subjected to seismic excitation [10–15]. However, among these solutions, friction dampers stand out due to their exceptional energy dissipation efficiency and ease of integration into building systems. Their ability to minimize structural and non-structural damage during moderate to severe seismic events has been extensively validated through experimental research [16–25] Furthermore, friction dampers are particularly appealing due to their straightforward implementation, cost-effectiveness, and independence from external power sources, making them a reliable choice for passive structural control strategies [26–33]

The rotational friction damper (RFD), first introduced by Mualla and Belev [34], demonstrated significant potential in enhancing structural behavior against seismic forces, as evidenced by experimental and numerical analyses conducted on single-story frames. Subsequent research expanded on the applications of RFDs in enhancing seismic performance.

The innovative concept of the rotational friction damper (RFD), which was initially presented and discussed by the scholars Mualla and Belev in their seminal work [34], has shown remarkable promise in significantly improving the structural response and behavior of various architectural frameworks when subjected to the adverse effects of seismic forces, a fact that has been substantiated through a combination of both experimental and numerical analyses meticulously conducted on single-story frame structures. In the wake of these pioneering findings, subsequent scholarly research has broadened the scope of applications for RFDs, particularly in relation to their potential contributions to enhancing seismic performance across a diverse range of structural configurations. For example, the research conducted by Patel and Jangid [35] delved into the intricate seismic response characteristics of adjacent buildings that are interconnected via the strategic installation of RFDs, thereby offering valuable insights into their collective behavior during seismic events. In a similar vein, Kim and colleagues [36] undertook a comprehensive evaluation of the effectiveness of RFDs, rigorously assessing their capacity to improve the overall seismic response and behavior of various types of structural systems, thereby contributing to the foundational knowledge in this critical area of civil engineering. Furthermore, Kaur and colleagues [37] performed an in-depth analysis focused on the seismic performance of mid- to high-rise buildings that have been equipped with friction dampers, placing particular emphasis on their significant role in alleviating earthquake-induced deformations and forces that can compromise structural integrity and safety. Additionally, Choi and Kim [38] explored the integration of frictional hysteretic energy dissipation mechanisms into reinforced concrete shear walls, which notably improved ductility under seismic loading. To address energy dissipation at low-frequency excitations, Sanati and colleagues [39] developed the rotational friction viscoelastic damper (RFVD), an enhanced RFD variant incorporating viscoelastic materials to improve energy dissipation under low-intensity vibrations. In order to assess seismic performance of buildings, Rayegani and colleagues [2] used four-joint RFDs (4J-RF) to enhance seismic resilience of buildings. The results of experimental and numerical analyses revealed that 4J-RF could efficiently dissipating energy and reducing residual displacements by up to 75%.

Subsequent advancements included Mirzabagheri et al.‘s [40] research, which demonstrated that multi-unit RFD configurations achieved superior energy dissipation compared to single-unit systems. Bonchev and colleagues [41] introduced RFDs as seismic links for retrofitting SMRFs, effectively replacing traditional link elements to minimize damage during seismic events. Anoushehei and colleagues [42] conducted experimental studies to evaluate the cyclic performance of RFDs with different friction pad materials, providing insights into optimizing damper configurations. Furthermore, Nabid and colleagues [43] proposed a refined design methodology for friction wall dampers in RC frames to maximize energy dissipation efficiency. Extending this work, Nabid and colleagues [44] implemented a performance-based optimization framework for reinforced concrete frames equipped with friction dampers, achieving a more uniform distribution of damage across the building height by strategically adjusting the dampers’ slip load. Collectively, these investigations highlight the RFD’s versatility and reliability in enhancing seismic behavior across a diverse range of structural applications.

To effectively reduce seismic responses in structures, the design and optimal placement of passive dampers are critical [45,46]. Researchers have employed various methods to optimize damper configurations. Zhang and Soong [47] and Shukla and Datta [48] used sequential optimization for viscoelastic dampers in multi-story buildings. Moreschi and Singh [49] optimized friction dampers and metallic yielding elements simultaneously. Fallah and Honarparast [50] applied a genetic algorithm to optimize Pall dampers under two scenarios with uniform or unique slip loads. Miguel and colleagues [51] optimized the force and placement of friction dampers, while Kim and An [52] improved an existing building’s seismic performance by optimizing damper distribution. In addition, self-centering systems have been identified as one of the innovative approaches utilized to mitigate lateral loads, such as those induced by earthquakes, in structural systems. A broad spectrum of previous studies has been dedicated to examining these so-called systems [53–55].

Jarrahi and colleagues [56] introduced an optimized design of RFDs by Utilizing a particle swarm optimization algorithm to enhance the seismic performance of inelastic SMRFs. Results indicate substantial reductions in roof displacement and hysteretic energy, confirming the RFD’s efficacy under various earthquake records. In another study, they developed a hybrid optimization framework combining binary and real-coded particle swarm optimization to determine the optimal placement and parameters of RFDs in nonlinear SMRFs. By addressing energy dissipation efficiency and structural drift constraints, their study demonstrates that a strategically reduced number of RFDs can significantly mitigate seismic responses without imposing excessive costs [56]. Moreover, Jarrahi and colleagues [57] also evaluated SSI effects on the seismic performance of SMRFs equipped with optimal RFDs. In this study, optimizing the placement and parameters of RFDs for enhance seismic performance of SMRFs have been discussed. The outcomes highlighted the remarkable impact of SSI on energy dissipation and structural behavior under seismic loading.

During an earthquake, the base movement of a building differs from free-field ground motion due to soil deformations beneath the structure, influencing its seismic responses. The structural behavior is directly influenced by the underlying soil as a result of the interaction between the structure and the soil [58,59]. This phenomenon, known as SSI, becomes significant when the underlying soil is not very stiff or solid rock [60,61]. Structures on deformable soils exhibit seismic responses distinct from fixed-base assumptions commonly used in practice [62,63]. Traditionally, the effects of SSI have been considered advantageous [64,65]. However, recent studies have demonstrated that SSI can also produce detrimental impacts [66]. Consequently, incorporating SSI effects is crucial for accurate seismic evaluations.

Some prior investigations assessed the influence of SSI on buildings equipped with dampers. In this context Araz and Elias [67] optimized tuned mass dampers (TMDs) configurations for seismic vibration control, accounting for SSI. Results demonstrate improved performance on soft soils, with reductions in displacements and accelerations under near and far-fault earthquakes. Also, the influence of key SSI parameters on the seismic efficiency of TMDs have been evaluated by Araz [68]. Numerical simulations on various building heights and soil conditions demonstrate that SSI significantly affects TMD performance, with reduced efficiency in low-rise structures on soft soils. Furthermore, soil nonlinearity effects on TMDs using a 3D FEM model have been analyzed by Wu and colleagues [69]. Results show that SSI reduces TMD effectiveness, especially in soft soils, while soil nonlinearity improves performance. Optimizing TMD parameters based on the soil-structure system frequency enhances seismic control compared to fixed-base design methods. Moreover, many other studies have analyzed SSI impacts on structural seismic behavior [70–74].

This investigation is primarily aimed at undertaking a comprehensive sensitivity analysis that pertains to the intricate design parameters associated with the yielding dampers, known as RFDs, which are specifically devised for the seismic protection SMRFs [75–77]. This study presents a novel contribution by conducting a comprehensive sensitivity analysis of the key parameters governing rotational friction dampers and enhancing their performance through parameter optimization, explicitly incorporating the influence of soil-structure interaction. Following this initial phase, the research proceeds to introduce a complex optimization problem that is fundamentally based on the seismic energy paradigm, with the objective of precisely determining the optimal configurations and specifications of the RFDs for both 6- and 10-story SMRFs when they are subjected to the dynamic forces generated by an artificial earthquake record. Within this analytical framework, the configuration of the RFDs, in conjunction with their frictional moment denoted as (M_f_) and the length of their vertical rigid beam represented by (h_a_), are meticulously identified as critical design variables, while the ratio that quantifies the maximum input energy relative to the maximum cumulative energy dissipated by the RFDs is systematically designated as the principal objective function of the optimization process. In order to effectively tackle the inherent challenges associated with this optimization task, a sophisticated hybrid methodology that integrates both binary and real-coded modified particle swarm optimization techniques, abbreviated as BRPSO, is employed to ensure a robust solution. Ultimately, the overall performance and efficacy of the SMRF that has been equipped with RFDs, which have been meticulously designed and optimized, is rigorously evaluated by incorporating the significant effects of soil-structure interaction (SSI) in response to four widely recognized real earthquake excitations, thereby facilitating a thorough understanding of the system’s behavior under seismic loading conditions. This comprehensive approach not only enhances the resilience of the structures but also contributes valuable insights into the field of earthquake engineering, particularly in the domain of structural optimization. The findings of this research endeavor are anticipated to have profound implications for the future design and implementation of seismic protection systems within the context of modern engineering practices.

## 2. Rotational friction dampers

**Fig 1:**
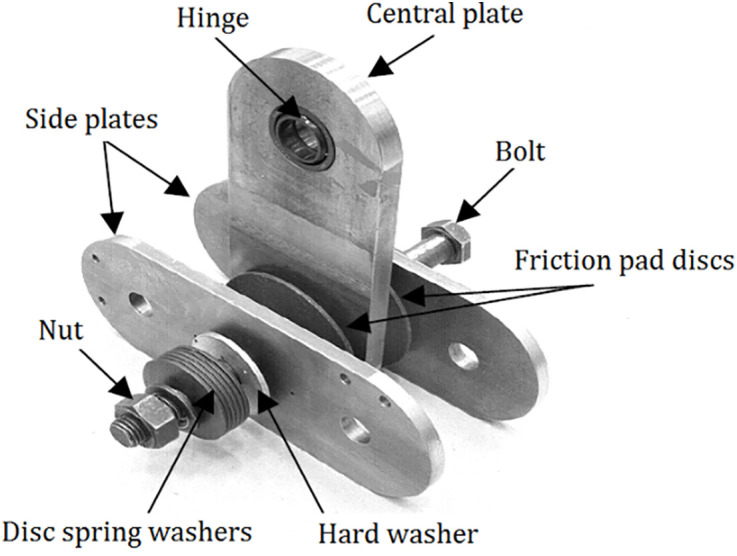
Specifications of the RFDs setup suggested by [34].

The various components that collectively constitute the system of RFDs, as illustrated comprehensively in Fig 1, encompass a pair of circular discs that are equipped with specialized friction pads, a unique central plate that is oriented vertically and positioned at the core, as well as two horizontal plates that extend laterally on either side. This intricate apparatus is meticulously attached to the central region of the beam via the aforementioned central plate, while the outermost boundaries of the lateral plates are firmly anchored to the supporting braces that provide stability. These braces are designed with the advanced capability of being pre-tensioned, a feature that is crucial in preventing any potential buckling phenomena when the structure is subjected to significant compressive forces that may arise during operational conditions. Thus, the effective interplay between these elements ensures the structural integrity and operational efficiency of the entire system under varying loads and stresses.

**Fig 2:**
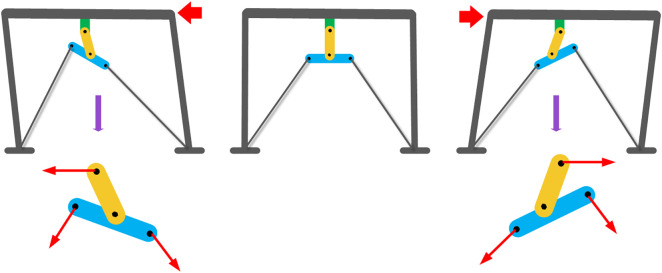
The RFD’s behavior mechanism (2002).

The frictional forces that are generated at the interface between the friction pads and the plates, when considered alongside the functionality of the bracing system, work in concert to effectively mitigate any potential movement of the frame during occurrences of seismic activity, thereby enhancing the overall stability and durability of the structure in question. Additionally, the operational dynamics of the RFDs are depicted in detail in Fig 2, which serves to highlight its remarkable capacity to dissipate energy efficiently, while simultaneously augmenting the seismic performance of the entire structural system, thereby ensuring greater resilience against the forces exerted during such events. Consequently, the integration of these mechanisms not only contributes to the immediate response of the structure under duress but also plays a pivotal role in the long-term sustainability and safety of the built environment in the face of unpredictable seismic occurrences.

The fundamental design parameters that are critical for the effective functionality of a RFD as well as its corresponding bracing system encompass several important elements, namely the frictional moment (M_f_) which plays a pivotal role in the energy dissipation characteristics of the system, the pre-stressing force (F_p_) that is meticulously applied to the bars to ensure adequate structural integrity and performance under various loading conditions, the cross-sectional area (A_b_) of the bars which directly influences the load-bearing capacity and overall stiffness of the damper system, and lastly, the dimensions pertaining to the length of both the central and side rigid plates (h_a_) which are essential in determining the overall geometric configuration and effectiveness of the bracing system in mitigating dynamic forces during seismic events. The detailed design principles and methodologies of the RFD have been thoroughly studied by Nielsen and Mualla [78]. Additionally, the relationships and equations governing the design process are comprehensively presented in Mualla [79], providing essential insights for the effective application of this damping system.

## 3. Sensitivity analysis

In this section, the model described in the Mualla [34] article in the previous part is subjected to sensitivity analysis, and the influence of parameters M_f_, F_p_, h_a_, and r on system behavior is studied. Initially, the parameters h_a_ (length of the vertical plate of the damper) and “r” (half the length of the horizontal plate of the damper) are considered as variables. The values of h_a_ are selected from 0.1, 0.2, 0.3, 0.4, and 0.5, while the values of “r” are chosen from 0.0826, 0.1652, 0.2478, 0.3304, and 0.4130. It is evident that the matrix formed by h_a_ and “r” constitutes a 5 × 5 matrix, where the diagonal elements represent the concentric installation configuration, and the remaining elements denote the eccentric installation configuration. For instance, when h_a_ = 0.4 and r = 0.3304, the system is concentric; for other possible values of r, the system becomes eccentric. It is noteworthy that a concentric system is defined as one where the intersection of the brace axes passes through the connection point of the damper and frame beam. Conversely, when this intersection occurs above or below the damper-beam connection point, the system is classified as eccentric [78].

**Fig 3:**
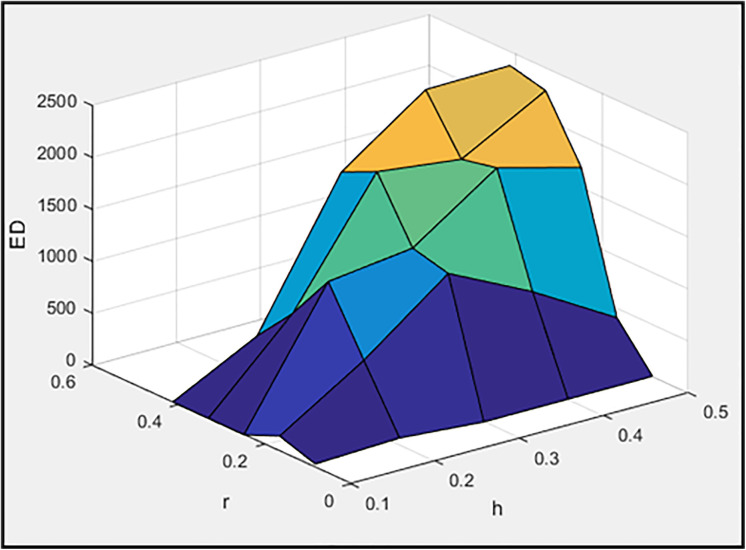
The effect of h_a_ and r values on dissipated energy.

**Fig 4:**
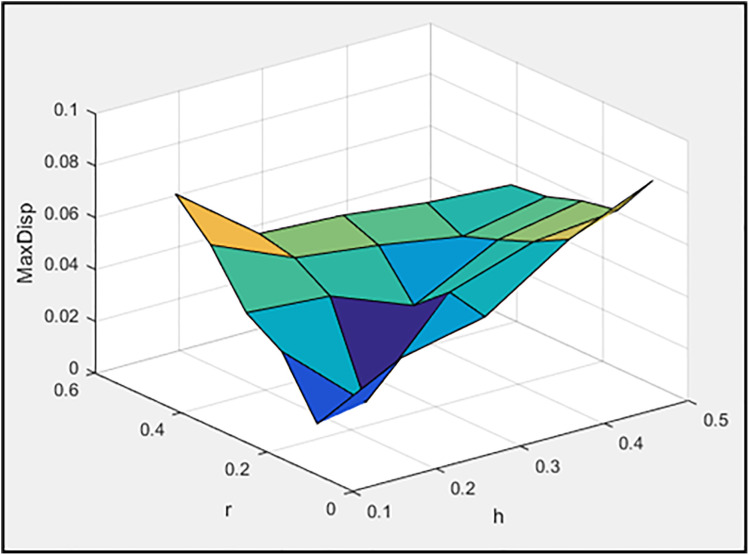
The effect of h_a_ and r values on maximum displacement of frame.

**Fig 5:**
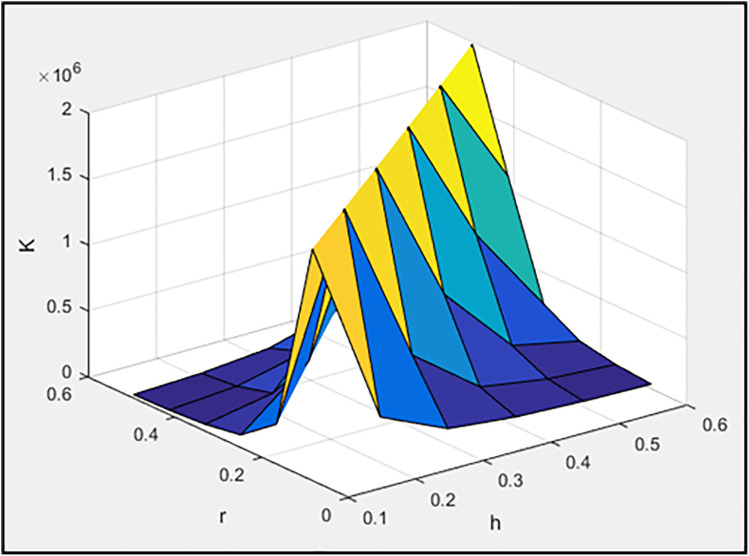
The effect of h_a_ and r values on the stiffness of the damper and brace system.

Figs 3–5 illustrate, respectively, the effect of damper geometry (h_a_ and r) on the energy dissipation of the damper (ED), the maximum horizontal displacement of the frame (MaxDisp), and the stiffness of the damper and braces system (K). All units in these figures are expressed in kgf-m.

Based on Fig 3, it is evident that the maximum energy dissipation occurs in the concentric installation configuration. Moreover, increasing the length of the damper plates enhances the dissipated energy. Also, the minimum horizontal displacement of the frame is achieved in the concentric installation configuration, as shown in Fig 4. Notably, the least displacement is observed at h_a_ = 0.2 and r = 0.1652, values that are neither the maximum nor minimum of their respective ranges. Consequently, it cannot be stated as a general rule that increasing or decreasing these two parameters will always reduce the maximum frame displacement. Instead, an optimal range for these parameters must be identified. Moreover, Fig 5 clearly demonstrates that the concentric installation configuration results in the highest stiffness for the damper and braces system. Additionally, increasing the length of the damper plates leads to an increase in system stiffness. As mentioned in the introduction, the governing parameters (including stiffness) for moment frames with chevron braces equipped with these dampers can be found in reference [78].

**Fig 6:**
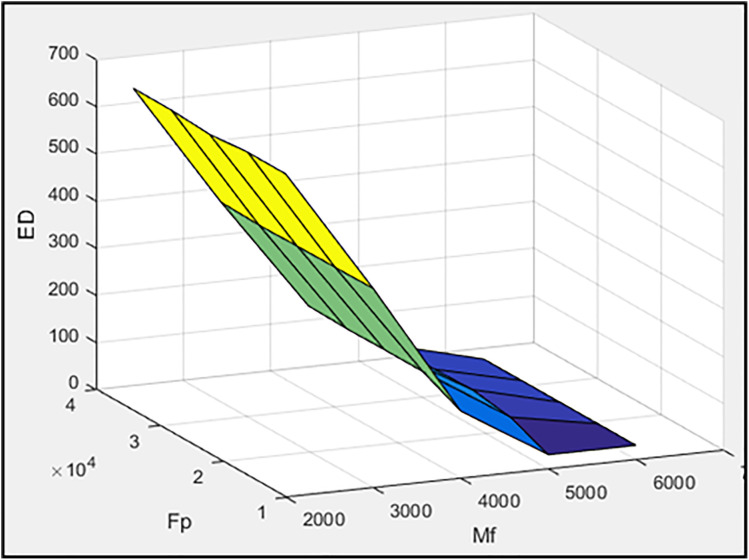
The effect of M_f_ and F_p_ values on dissipated energy.

**Fig 7:**
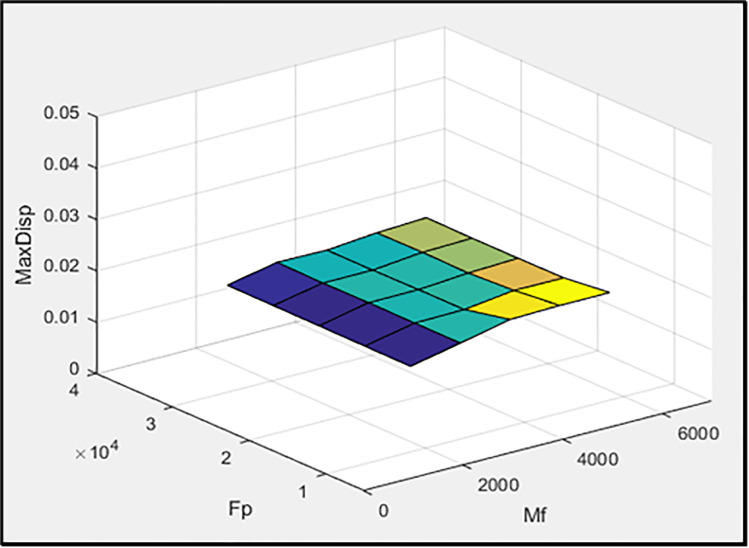
The effect of M_f_ and F_p_ values on maximum displacement of frame.

**Fig 8:**
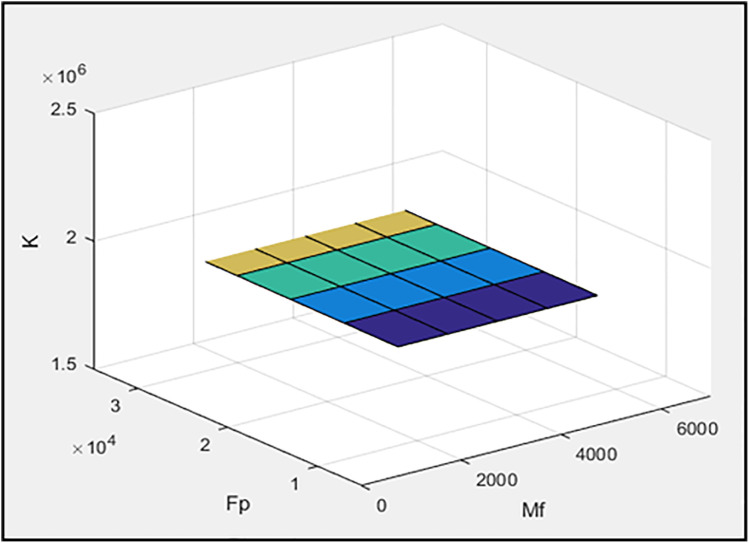
The effect of M_f_ and F_p_ values on the stiffness of the damper and brace system.

In the second stage, h_a_ and r are set to 0.2 and 0.1652, respectively, while the parameters M_f_ (slip friction moment) and F_p_ (pre-tension force of the braces) are considered as variables. The values of M_f_ are selected from 2000, 3,000, 4,000, 5,000, and 6,000, while the values of F_p_ are defined as six times the corresponding M_f_ values. Figs 6–8 illustrate the effects of M_f_ and F_p_ on the energy dissipation of the damper (ED), the maximum horizontal displacement of the frame (MaxDisp), and the stiffness of the damper and braces system (K), respectively. All units in these figures are expressed in kgf-m. Fig 6 clearly demonstrates a pronounced inverse relationship between the slip friction moment and the amount of energy dissipated (ED). As M_f_ increases, the system’s ability to dissipate energy diminishes markedly. This is primarily due to the fact that a higher slip friction moment restricts the damper from transitioning into its sliding phase, which is essential for energy dissipation through friction. Conversely, F_p_ appears to exert only a marginal influence on energy dissipation, suggesting that it is not a primary controlling parameter in this context. Fig 7 presents the variation of the maximum displacement of the frame with respect to M_f_ and F_p_. Although there are slight changes in displacement values with increasing M_f_ and F_p_, the overall trend remains relatively flat. This implies that neither parameter has a substantial impact on frame displacement, which could be attributed to the fact that displacement is more sensitive to system-level dynamics rather than isolated damping or bracing characteristics. Fig 8 sheds light on the influence of M_f_ and F_p_ on the stiffness of the damper-brace system (K). As illustrated, variations in these parameters do yield slight modifications in the overall stiffness. However, much like in the case of displacement, the observed changes are not significant. This again indicates that the system’s stiffness is relatively robust against moderate changes in either slip moment or pre-tension force.

## 4. Seismic energy approach

The ability of a structural system to withstand seismic events is contingent on its capacity to dissipate seismic energy through various mechanisms, including kinetic energy, damping energy, inelastic hysteresis, and elastic strain energy [80–84]. The incorporation of RFDs enhances this dissipation through frictional mechanisms, thereby improving the system’s overall energy absorption efficiency. The governing equations describing structural motion and energy distribution have been extensively explored in previous studies [56,57,85]. In the present work, the methodology previously developed is employed to quantify input energy, cumulative hysteretic energy, and dissipated energy within RFD-integrated systems. The energy balance equation and associated formulations for kinetic, damping, and absorbed energy remain consistent with prior research, with additional refinements tailored to the specific characteristics of the current study.

## 5. Particle swarm optimization algorithm

Metaheuristic optimization algorithms have been widely utilized in structural optimization [86–95], with Particle Swarm Optimization (PSO) being a prominent example due to its adaptability to both continuous and discrete variables. Although the convergence speed of PSO is very slow towards the global optimum and is trapped by poor local minima. Hence, diverse variants of PSO algorithm were developed for overcoming its drawbacks.

Originally inspired by the coordinated movements of bird flocks, PSO iteratively refines candidate solutions until convergence criteria are met. The Real-Coded PSO (RPSO) and Binary-Coded PSO (BPSO) variants offer tailored approaches for different problem formulations, utilizing velocity and position updates to navigate the solution space. To enhance convergence and mitigate local optima entrapment, Modified PSO (MPSO) incorporates an additional stochastic perturbation factor. The mathematical foundations and implementation details of these algorithms have been extensively addressed in prior studies [56,57], forming the basis for their application in the present research.

## 6. Modeling assumptions

### 6.1. Modeling of RFD

The intricacies involved in the modeling processes and the subsequent verification of the RFD within the computational framework of OpenSees are thoroughly elucidated in the present section. The distinctive behavior of the frictional hinge was meticulously simulated through the implementation of a zero-length element that exhibited an elastic-perfectly plastic response, characterized by an exceptionally high initial stiffness that is critical for accurate modeling. In order to facilitate this simulation, two nodes possessing identical spatial coordinates were strategically defined at the location of the frictional hinge, and their translational degrees of freedom were effectively constrained to ensure proper functionality. The bracing bars, fundamental components of the structural system, were adeptly modeled utilizing the corotTruss element, which allows for the capture of the non-linear behavior associated with such elements. Furthermore, the pre-tensioned force present in the bracing bars was integrated into the model through the initial strain technique, which was operationalized via the innovative use of InitStrainMaterial, thus ensuring that the behavior of the bars under load was accurately represented. To substantiate the efficacy of the RFD modeling approach employed in OpenSees, a rigorous validation process was undertaken, wherein an elastic single-story SMRF that was equipped with an RFD, previously investigated by Mualla and Belev [34], was meticulously considered within the broader scope of the study conducted by Jarrahi et al. [56,57,96]. This comprehensive validation was essential to ensure that the modeling techniques accurately reflected the physical behavior of the system under various loading conditions. The careful attention to detail in modeling components such as the frictional hinge and the bracing bars serves to enhance the reliability of the computational results, thereby contributing to a more profound understanding of the RFD’s impact on structural performance.

### 6.2. Modeling of an SMRF

**Fig 9:**
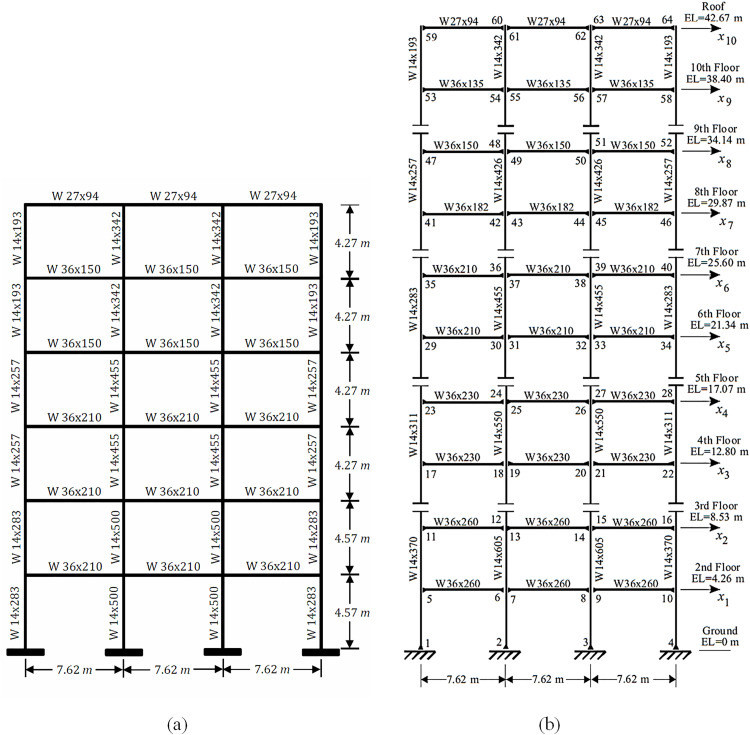
Characteristic of 6- and 10-story SMRF [99,98].

The computational analyses conducted in this research are carried out on 6- and 10-story SMRF, which were established as a reference structure by Wong [97] and subsequently by Wong and Johnson [98]. The characteristics of the structural framework are depicted in Fig 9.

**Fig 10:**
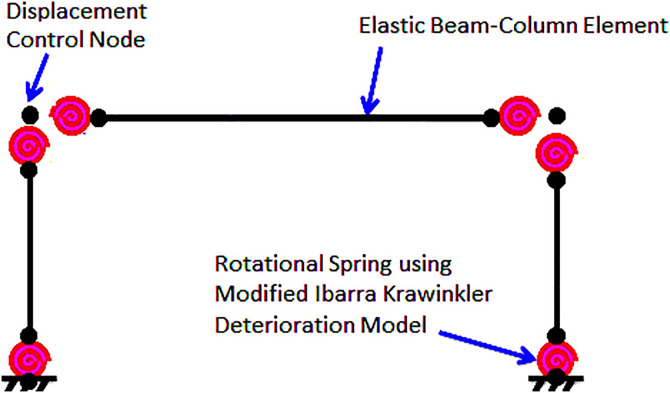
The SMRF characterized by lumped plasticity within the OpenSees.

**Fig 11:**
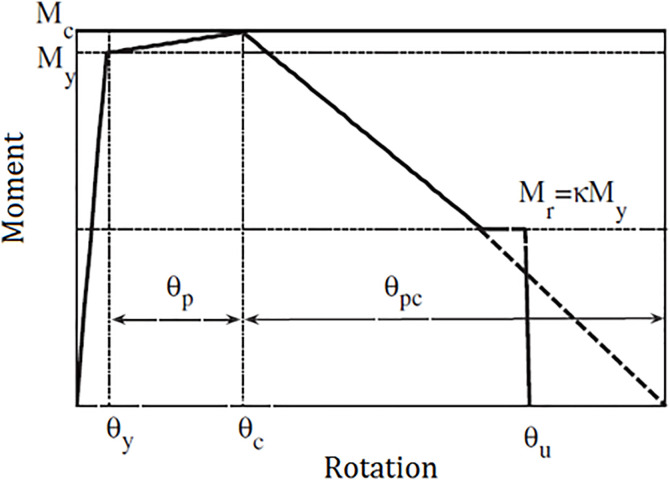
The monotonic response curve of the modified IMK [100,101].

The total mass associated with each individual story, along with the uniformly distributed load acting upon the beams of the 6-story SMRF, was meticulously assumed to be a significant value of 2,942 kN, while the uniformly distributed load was designated to be 21.89 kN/m, thereby establishing a foundational parameter for the structural analysis. In a parallel manner, when considering the 10-story SMRF, these crucial parameters were respectively taken as a total mass of 2,147 kN and a uniformly distributed load of 21.89 kN/m, ensuring consistency across the modeling framework. Furthermore, a damping ratio of 3% was uniformly adopted for both structural models, reflecting a common practice in structural dynamics to account for energy dissipation mechanisms. In addition, the yield stress (Fy) of the structural steel was precisely defined at a value of 248.2 megapascals (MPa), while the modulus of elasticity (E) for the steel was established at a value of 2 × 10^5^ megapascals (MPa), which are both critical parameters in determining the material behavior under various loading conditions. The lumped plasticity model was effectively implemented within the analysis framework by utilizing zero-length plastic hinges strategically located at the ends of the elastic beam-column elements, as meticulously illustrated in the accompanying Fig 10. To accurately capture the bilinear hysteretic response characteristic of these structural elements, the Ibarra–Medina–Krawinkler (IMK) model [100] was employed, with its pertinent properties comprehensively depicted in Fig 11 for further clarity. It is important to note that both aforementioned frames have undergone rigorous validation in prior research conducted by Jarrahi and colleagues, as referenced in studies [56,57,85].

### 6.3. Soil-structure interaction (SSI) modeling procedure

**Fig 12:**
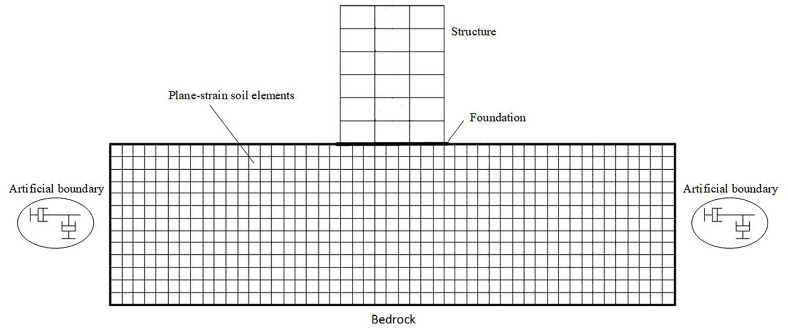
Fithe fnite element modeling framework developed for the SSI system, implemented using the OpenSees simulation platform.

SSI plays a critical role in influencing structural behavior, especially in buildings equipped with damping systems [102]. In this study, SSI was modeled using the direct finite element approach developed by Jaya and Prasad [103]. This method enables the simultaneous modeling of both the soil and structural components, thereby facilitating a detailed assessment of their nonlinear dynamic behavior using OpenSees simulations. The finite element configuration of the SSI system is depicted in Fig 12.

**Fig 13:**
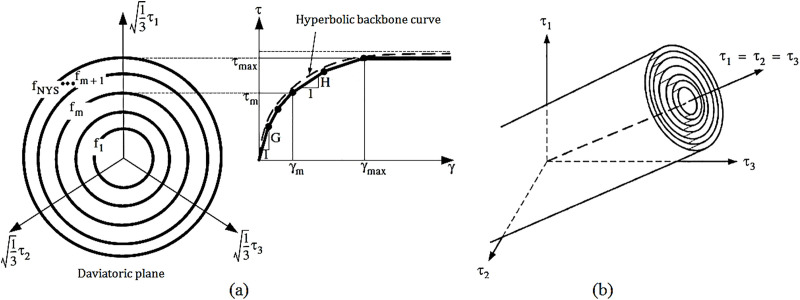
Illustration of the multi-yield-surface formulation based on the J2 plasticity theory: (a) Octahedral representation of shear stress–strain response; (b) multi-surface Von Mises yield criteria [105].

Rayhani and Naggar [104] experimentally examined the impact of varying horizontal soil boundary distances on the seismic response of different structural systems. Their analysis considered two boundary distances, 10B and 5B, where *B* denotes the foundation width. The results revealed that increasing the boundary distance from 5B to 10B produced only a marginal 5% change in seismic response, while significantly reducing computational cost and processing time. Furthermore, their findings confirmed that seismic wave amplification is primarily concentrated within the upper 30 meters of the soil profile, consistent with contemporary seismic design codes, which base local site effect assessments on the properties of the top 30 meters of soil. Based on these observations, the current study adopted a horizontal soil boundary distance of five times the structure’s width (4,500 inches) and assumed a soil domain depth of 30 meters. The soil domain was modeled using isoparametric four-node quadrilateral elements under plane strain conditions, with a constant out-of-plane thickness equivalent to the inter-frame spacing. Fig 13 presents the modified pressure-independent multi-yield J2 plasticity model introduced by Zhang and colleagues [105], with parameters calibrated according to the guidelines specified in the OpenSees manual.

To simulate radiation damping and prevent the artificial reflection of outward-propagating shear and dilatational waves, Lysmer–Kuhlemeyer dashpots, originally proposed by Lysmer and Kuhlemeyer [106], were applied at the free-field boundaries of the soil domain. These dashpots, illustrated in Fig 12, function by absorbing seismic energy at the model boundaries. In this study, the dashpots were implemented using zero-length elements assigned with a viscous uniaxial material model. For a comprehensive explanation of their formulation and mechanical behavior, readers are referred to Lysmer and Kuhlemeyer [106]. The raft foundation was idealized as rigid, and the interface between the foundation and the surrounding soil was modeled using nodes with appropriate constraints. To ensure compatible displacement between the soil and foundation in both horizontal and vertical directions, the *equalDOF* command was employed in the X and Y directions. The primary soil material properties used in the numerical simulations are listed in Table 1, while supplementary parameters were derived from shear wave velocity (Vs) and Poisson’s ratio (ν), following the guidelines provided by Khatibinia and colleagues [73].

**Table 1:** Summary of the governing parameters and constitutive criteria adopted in the development of the pressure-insensitive multi-yield plasticity formulation.

Parameter	Description	Value
$V_{s}\;(m/s)$	Shear wave velocity	275
$\rho \;(ton/m^{3})$	Mass density	1.7
v	Poisson’s ratio of soil	0.35
ϕ	Friction angle at peak shear strength	24
ζ	viscous damping	10%

## 7. Optimization of placement and parameters of RFDs for seismic protection of SMRF

This section meticulously delineates the concurrent optimization processes that are employed in the strategic placement as well as the intricate design parameters of RFDs, which are specifically intended for the enhancement of seismic protection measures applicable to SMRFs.

### 7.1. Problem optimization

In this context, the location vector of the RFDs (P) is characterized as an N_p_-dimensional binary vector, wherein each component assumes a value of either 1 or 0, signifying the existence or non-existence of a damper, respectively. Considering that the upper limit of dampers that can be integrated into the SMRF is represented by N_d_, the vector P delineates the various potential configurations for damper placement within the structure. For the N_d_ dampers, a design vector that encompasses the frictional moment (M_f_) and the vertical rigid beam length (h_a_) is proposed, with both being regarded as continuous design variables. Consequently, all design variables are aggregated into a (N_p_ + N_d_ × 2)-dimensional vector (i.e., X = {P, M_f_, h_a_}). The optimization challenge is constrained by a limit on the maximum inter-story drift of the structure. The objective function is articulated as the maximum seismic input energy (E_i_) that penetrates the structure, normalized by the maximum cumulative energy dissipated within the RFDs (E_RFDs_). Thus, the concurrent optimization of RFD distribution and design parameters can be articulated as follows:


$$\begin{array}{c} Find:\;\;\;\;\;\;\;\;\;\;\;X=\{P,M_{f},h_{a}\}\\ Minimize:f(X)=\frac{E_{i}(X)}{E_{RFDs}(X)}\\ subject\;to:\delta (X)\leq \delta_{all}\\ \;\;\;\;\;\;\;\;\;\;\;\;\;\;\;\;\;\;\;\;\;\;\;h_{a,\min}^{i}\leq h_{a}^{i}\leq h_{a,\max}^{i},\;i=1,2,...,N_{d}\\ \;\;\;\;\;\;\;\;\;\;\;\;\;\;\;\;\;\;\;\;\;\;M_{f,\min}^{i}\leq M_{f}^{i}\leq M_{f,\max}^{i},\;i=1,2,...,N_{d} \end{array}$$ (1)

where ${\text{h}}_{\text{a},\text{min}}^{\text{i}}\;$ and ${\text{h}}_{\text{a},\text{max}}^{\text{i}}\;$ denote the lower and upper limits of the vertical rigid beam length associated with the ith damper, respectively; ${\text{M}}_{\text{f},\text{min}}^{\text{i}}$ and ${\text{M}}_{\text{f},\text{max}}^{\text{i}}\;$ represent the lower and upper limits of the frictional moment attributed to the ith damper, respectively. is the permissible threshold of the inter-story drift. In order to integrate constraints within the optimization framework, Coello [107] undertook an extensive investigation into techniques for handling constraints. Notably, the external penalty function has emerged as one of the most prevalently utilized methodologies in structural optimization [108–110]. By employing this strategy, the constrained optimization challenge associated with the RFD can be reformulated into an unconstrained format. This technique articulates the penalized objective function in the following manner:


$$\tilde{f}(X)=f(X)\left(1+r_{p}P_{f}\right)$$ (2)

where P_f_ is the total penalty; and r_p_ is an adjusting coefficient introduced by Chen and Chen [108] as:


$$r_{p}=r_{p1}\left[1+0.2(l-1)\right]\leq 4r_{p1}$$ (3)

where r_p1_ is an initial adjusting value in the first iteration and l is the generation counter. It is noted that the selection reason of penalty coefficient and their sensitivity to optimization performance have been discussed in the previous studies such as Rajeev and Krishnamoorthy [111].

### 7.2. *Optimization of placement and parameters* of *RFDs*

**Fig 14:**
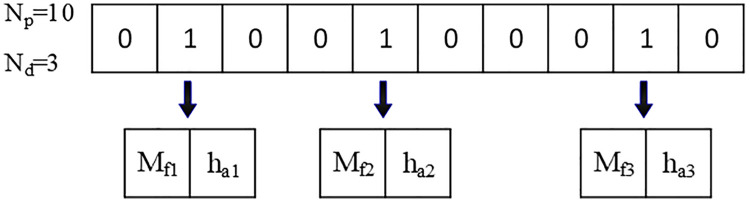
the functioning of BRPSO.

In the context of this research, a novel hybrid optimization algorithm has been meticulously crafted, which integrates both binary and real-coded modified Particle Swarm Optimization (PSO) techniques. This innovative approach, which has been designated as BRPSO, has been specifically designed to concurrently optimize not only the strategic placement but also the critical parameters of the RFDs utilized within the framework of the 6- and 10-story SMRF depicted in Fig 9. To facilitate this intricate optimization process, the maximum permissible quantity of RFDs that can be incorporated into the structural design has been formally denoted by the symbol N_d_. In pursuit of ascertaining the most advantageous placement of the RFDs, each particle within the BRPSO algorithm has been assigned a binary string, referred to as P, which comprises a total of N_p_ bits. It is noteworthy that, in this specific formulation, N_p_ has been established as 10, which directly correlates to the total number of stories present within the structural configuration being analyzed. Within this binary representation system, a value of 1 located at the i-th position of the binary string P unequivocally indicates the presence of an RFD positioned at the i-th story level, while conversely, a value of 0 signifies the complete absence of an RFD at that particular elevation. Progressing to the subsequent phase of the optimization process, a N_d_ × 2-dimensional vector has been assigned to each particle, which serves the purpose of encapsulating the essential parameters associated with the RFDs that have been installed. It is crucial to emphasize that these specific parameters are exclusively linked to the positions within the binary string P where the value has been identified as 1, thus ensuring a direct correlation between the binary representation and the physical attributes of the dampers. The comprehensive optimization procedures, alongside the intricate encoding strategy employed within the BRPSO framework, are visually represented and elucidated in detail within Fig 14, thereby providing a clearer understanding of the methodological advancements achieved through this research initiative.

## 8. Numerical results of optimization

The most advantageous arrangement of RFDs, in conjunction with the associated parameter values that correspond to these distributions, was meticulously executed for frames characterized by 6- and 10-stories in the context of SMRFs. In addition, the established upper and lower bounds concerning the design variables that are relevant to the RFDs are comprehensively detailed and presented in Table 2, which serves as a critical reference point for understanding the constraints and limits imposed on the design process.

**Table 2:** Defined Ranges for RFD Parameter Values in 6- and 10-Story SMRFs.

RFD parameter	SMRF	Lower bound	Upper bound
*M*_*f*_ (Kips.in)	6-Story	200	3,000
	10-Story	300	4,000
*h*_*a*_ (in)	6-Story	5	25
	10-Story	5	25

**Fig 15:**
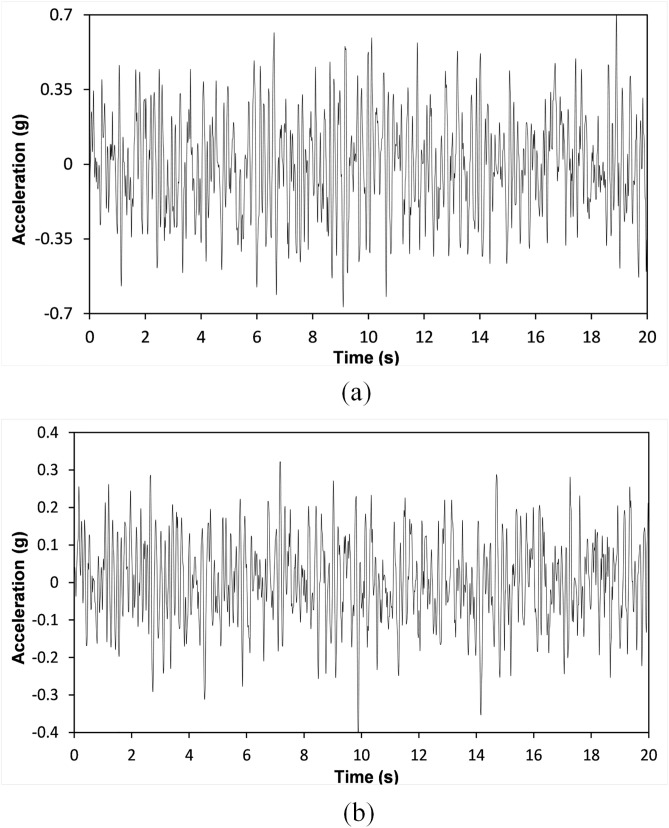
Time history of artificial record with a PGA of (a) 0.7g and (b) 0.4g.

In the present research, the parameter values for the RBPSO were established in accordance with the comprehensive guidelines outlined by Gharehbaghi and Khatibinia [87]. Consequently, the upper limit for the number of iterations (lmax) and the size of the population (N) were designated as 100 and 30, respectively. The minimum and maximum weight coefficients (i.e. *ω*min and *ω*max) and were allocated values of 0.1, 0.4, and 25, correspondingly. Moreover, the optimal configuration for the RFDs along with their associated parameter values were initially computed for the SMRF subjected to a simulated earthquake event. The synthetic seismic record was generated utilizing the well-established Kanai–Tajimi methodology. This technique conceptualizes seismic excitation as a stationary process, wherein the input motion is depicted as a white noise signal. The power spectral density (PSD) function of the excitation is articulated as follows:


$$S_{K.T}(\omega )=S_{0}\left[\frac{{\omega_{g}}^{4}+4{\zeta_{g}}^{2}{\omega_{g}}^{2}\omega^{2}}{(\omega^{2}-{\omega_{g}}^{2}{)}^{2}+4{\zeta_{g}}^{2}{\omega_{g}}^{2}\omega^{2}}\right];S_{0}=\frac{0.03\zeta_{g}}{\pi \omega_{g}(4{\zeta_{g}}^{2}+1)}$$ (4)

where S_0_ denotes the invariant spectral density. Furthermore, the frequency and the fundamental damping coefficient are postulated to be 25.13 rad/s (equivalent to 4 Hz) and 0.8, respectively, in accordance with the findings of Wu and colleagues [112] for Site Class C, as delineated in ASCE 7-16 [64]. The PGA of the synthetically generated earthquake employed in the optimization procedure was established at 0.7g for the 6-story framework and 0.4g for the 10-story configuration. Additionally, Fig 15(a) and (b) depict the temporal progression of the aforementioned synthetic seismic records.

A total of five separate optimization executions were conducted, resulting in the identification of the optimal solution that produced the minimum value for the objective function, which was recognized as the global optimum for the RFDs. This study examined and determined the ideal configuration of the RFDs along with their associated parameter values for varying N_d_ in both 6- and 10-story structural frameworks. The optimal arrangement of the dampers, along with the corresponding values of the objective function, is presented in Table 3.

**Table 3:** Optimal Damper Configurations and their Associated Objective Function Values.

*N*_*d*_	6-Story		10-Story	
	Best placement	Objective function	Best placement	Objective function
1	[0,0,0,0,0,1]	2.185	[0,0,0,0,0,0,0,0,0,1]	4.767
2	[0,0,0,0,1,1]	1.952	[0,0,0,0,0,0,0,0,1,1]	3.834
3	[1,0,0,1,0,1]	1.702	[1,0,0,0,0,0,1,0,0,1]	2.993
4	[1,0,0,1,1,1]	1.406	[1,0,0,1,0,1,0,0,0,1]	2.416
5	[1,0,1,1,1,1]	1.194	[1,0,0,1,0,1,0,1,0,1]	2.053
6	[1,1,1,1,1,1]	1.183	[1,0,1,0,1,0,0,1,1,1]	1.755
7			[1,0,1,0,0,1,1,1,1,1]	1.511
8			[1,1,0,1,1,1,1,1,0,1]	1.508
9			[1,1,0,1,1,1,1,1,1,1]	1.511
10			[1,1,1,1,1,1,1,1,1,1]	1.491

The optimization outcomes delineated in Table 3 indicate that an augmentation in the optimal RFDs within the SMRF framework leads to a reduction in the objective function. A comparative analysis of the objective function values demonstrates that a negligible difference is noted when the quantity of RFDs is increased from 5 to 6 for a 6-story frame. Furthermore, a similar trend is observed in the case of the 10-story structure, wherein the number of RFDs is altered from 7 to 10. Additionally, the optimal parameters for dampers pertaining to both the 6-story and 10-story frames are presented in Tables 4 and 5, respectively.

**Table 4:** Optimal RFD Parameter Values for Configuration in the 6-Story Frame.

Story	Total Number of RFDs					
	1	2	3	4	5	6
6	2711^a^	1801	1,253	796	2,781	824
	7.26^b^	15.49	16.72	17.55	22.21	11.61
5	–	2018	–	1986	2,540	1,774
		10.74		14.18	14.93	17.28
4	–	–	1,515	2,327	1,639	1,546
			17.14	17.02	21.43	10.50
3	–	–	–	–	2,662	1813
					14.26	20.35
2	–	–	–	–	–	2,719
						9.36
1	–	–	1,598	2,744	1990	2,840
			12.35	17.61	8.92	10.39

a: *M*_*f*_ (Kips.in), b: *h*_*a*_ (in).

**Table 5:** The optimal RFD parameter values for the allocation of RFDs in a 10-story frame.

Story	Total Number of RFDs									
	1	2	3	4	5	6	7	8	9	10
10	3971^a^	3,010	3,105	2,111	2,458	2,456	3,109	3,119	2,840	3,502
	8.23^b^	19.99	14.93	17.84	23.54	15.28	12.41	14.49	21.81	14.33
9		2,981				3,866	1,547		3,664	3,616
		16.41				11.98	14.44		23.41	11.93
8					2,147	2,159	3,547	3,857	3,311	2,715
					18.47	10.74	24.25	13.19	18.22	18.10
7			2,320				2,247	3,002	2,658	2,451
			18.74				18.11	7.37	19.18	13.63
6				1,159	1843		1,588	1815	484	1927
				16.41	15.29		17.58	19.81	6.92	18.82
5						2,921		2,120	2,525	2,256
						14.17		19.84	19.50	22.24
4				1,258	3,540			3,098	1,725	1,451
				16.32	22.31			17.56	21.09	11.24
3						2,451	1935			3,147
						17.13	21.02			13.13
2								1986	3,216	1,181
								18.31	17.48	14.36
1			1816	2,763	841	2,525	1984	2,471	2,432	3,221
			20.55	11.15	7.32	12.84	13.27	17.75	12.36	20.08

a: M_f_ (Kips.in), b: h_a_ (in).

In Table 6, one can observe a comprehensive illustration of the distinctive characteristics pertaining to the maximum input energy, the hysteretic energy, as well as the dissipated energy, which are all meticulously delineated for the structural framework that incorporates the optimally designed RFDs, and this detailed analysis is subsequently juxtaposed with the corresponding energy characteristics of the structural framework that does not integrate such RFDs, specifically focusing on a 6-story frame.

**Table 6:** Comparative analysis of maximum input, hysteretic, and dissipated energy in a 6-Story SMRF with various optimal RFD configurations.

Energy	Total Number of RFDs						
	0	1	2	3	4	5	6
*E*_*i*_ (Kips.in)	24,764	24,357 (1.64)^*^	24,003 (3.07)	23,519 (5.03)	22,143 (10.58)	21,408 (13.55)	21,312 (13.94)
*E*_*RFDs*_ (Kips.in)	–	11,148	12,295	13,821	15,754	17,925	18,012
*E*_*h*_ (Kips.in)	13,276	4,639 (65.06)^*^	3,237 (75.62)	2,218 (83.29)	1,271 (90.43)	238 (98.21)	199 (98.50)

* The percentage of reduction

**Fig 16:**
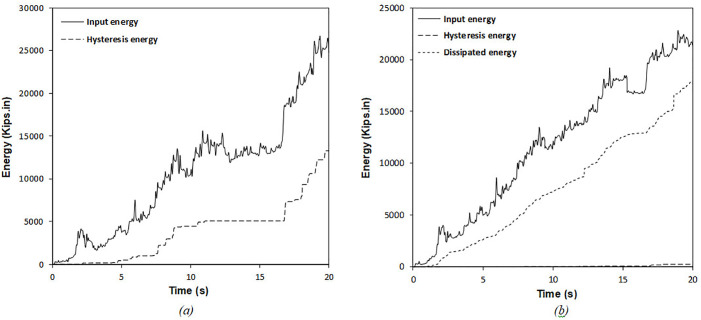
Energy time histories for the 6-story frame subjected to synthetic ground motion: (a) SMRF without RFDs and (b) SMRF with optimally configured RFDs.

It is clear from Table 6 that enhancing the number of dampers leads to a reduction in input energy, while the energy dissipated by the RFDs shows an upward trend. The results further clarify that optimizing the position and characteristics of the RFDs significantly diminishes the total hysteretic energy, thus mitigating seismic damage to the structure. The ideal results highlighted in Table 6 indicate that a substantial portion of the input energy is absorbed by the optimal RFDs, allowing for only a tiny fraction of the seismic input energy to be transferred to the structural components, ultimately resulting in less structural damage during seismic events. As shown in Table 6, increasing the number of RFDs from 5 to 6 corresponds to a reduction percentage in cumulative hysteretic energy of 98.21% and 98.50%, respectively. This pattern is also observed in input energy, with reduction percentages for the SMRF equipped with 5 and 6 RFDs documented at 13.5% and 13.94%, respectively. The dissipated energy values for the optimal configurations of 5 and 6 RFDs are recorded as 17,925 and 18,012 Kips.in, respectively. As a result, the significant change in dissipated energy when increasing the number of RFDs from 5 to 6 is not apparent. Therefore, the optimal number of dampers can be considered to be 5, and any further increase in the number of dampers may lead to substantial costs without providing a notable decrease in the structural seismic responses. Fig 16 displays the comparative evaluation of input, hysteretic, and dissipated energies during the simulated earthquake for the SMRF without RFDs compared to the SMRF fitted with the optimal RFDs.

**Fig 17:**
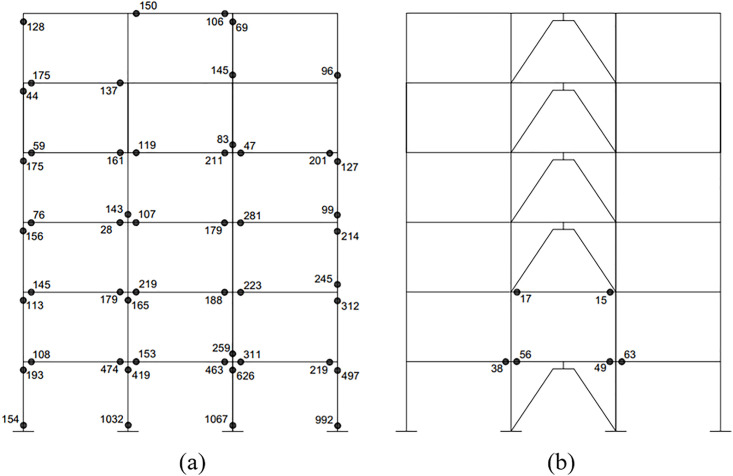
Comparison of hysteretic energy dissipation at each plastic hinge of the 6-story frame (a) without RFD (b) with optimal RFDs (unit: Kips.in).

The findings indicate that the hysteretic energy of the SMRF, when integrated with the optimal RFDs, is substantially reduced during the simulated seismic event. Consequently, the structural integrity is preserved against significant structural damage. Fig 17 presents a comparative analysis of the hysteretic energy dissipation at the plastic hinges of the structure outfitted with five optimal dampers in contrast to that of the structure devoid of RFDs. It is evident that a significant proportion of the structural components within the system equipped with the five optimal dampers remains within the elastic limits, thus protecting the structural frame from severe damage.

**Fig 18:**
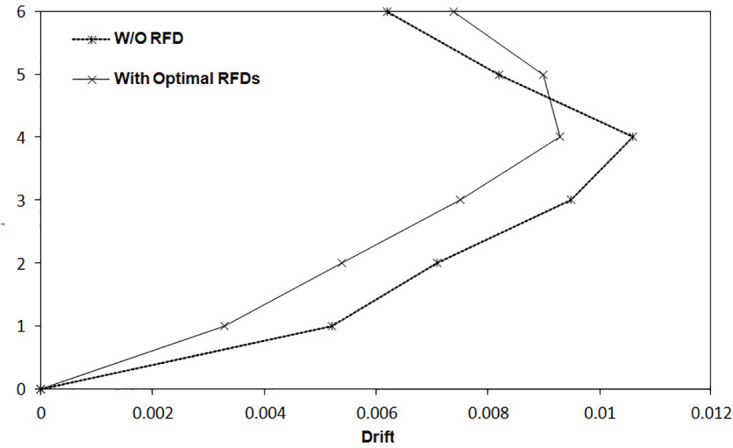
Comparative assessment of maximum structural drift with and without optimally designed RFDs.

Furthermore, Fig 18 illustrates the maximum story drift ratio for the 6-story SMRF both in the absence of RFDs and when equipped with the five optimized RFDs. As evident from the so-called figure, the incorporation of RFDs significantly reduces the inter-story drift ratio, demonstrating their effective performance. However, in the upper stories, the inter-story drift ratio of the frame with RFDs exceeds that of the frame without RFDs. This discrepancy arises because the optimization process is primarily energy-based, leading to a trade-off in drift reduction at higher levels.

Table 7 meticulously delineates the maximum input energy, the hysteretic energy, and the dissipated energy associated with the structural system that is equipped with the optimally designed and strategically placed RFDs, while simultaneously providing a comparative analysis with the corresponding energy metrics of the 10-story structural frame.

**Table 7:** Comparative evaluation of maximum input, hysteretic, and dissipated energy values in a 10-story SMRF with various optimally configured RFDs.

Energy	Total Number of RFDs										
	0	1	2	3	4	5	6	7	8	9	10
E_i_ (Kips.in)	14,413	14,073 (2.36)^*^	13,755 (4.57)	13,208 (8.36)	12,419 (13.83)	11,962 (17.01)	11,341 (21.31)	11,075 (23.16)	10,940 (24.10)	11,121 (22.84)	11,048 (24.76)
E_RFDs_ (Kips.in)	---	2,952	3,588	4,413	5,141	5,827	6,463	7,331	7,254	7,359	7,409
E_h_ (Kips.in)	3,542	3,283 (7.31)^*^	2,837 (19.90)	2,318 (34.56)	1,475 (58.36)	717 (79.76)	143 (95.96)	8 (99.77)	1 (99.97)	0 (100)	0 (100)

* The percentage of reduction

**Fig 19:**
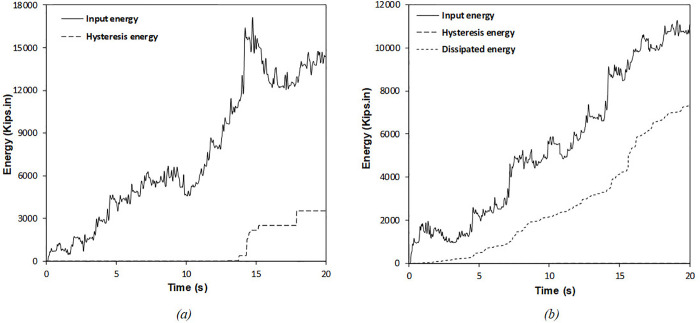
Time history of energy components in the 10-story frame subjected to the artificial record: (a) SMRF without RFD and (b) SMRF equipped with optimal RFDs.

As demonstrated in Table 7, an increase in the number of dampers results in a reduction of the input energy, while the energy dissipated in experiments involving the RFDs shows a progressive increase. The results further clarify that the deliberate optimization of the placement and characteristics of the RFDs significantly diminishes the total hysteretic energy, thus aiding in the reduction of seismic damage to the structure. The optimal results presented in Table 7 indicate that a considerable proportion of the input energy is absorbed by the strategically positioned RFDs, while only a small fraction of the seismic input energy is transmitted to the structural elements, which subsequently lessens the structural damage incurred during seismic activity. From Table 7, it is observable that increasing the number of RFDs from 7 to 10 results in a reduction in cumulative hysteretic energy of 99.97% and 100%, respectively. This pattern is similarly noted in the input energy, with the reduction percentages for the SMRF outfitted with 7 and 10 RFDs recorded at 23.16% and 24.76%, respectively. The dissipated energy values for the optimal configurations of 7 and 10 RFDs are quantified as 7,331 and 7,409 Kips.in, respectively. Thus, the significant alteration in dissipated energy upon increasing the RFDs from 7 to 10 is negligible. Therefore, the optimal count of dampers may be regarded as 7, as any further augmentation in the number of dampers incurs significant construction costs without providing a meaningful reduction in structural seismic responses. Fig 19 presents a comparative analysis of the input, hysteretic, and dissipated energies during the artificially simulated earthquake for the SMRF devoid of RFDs and the optimally equipped RFDs-SMRF.

**Fig 20:**
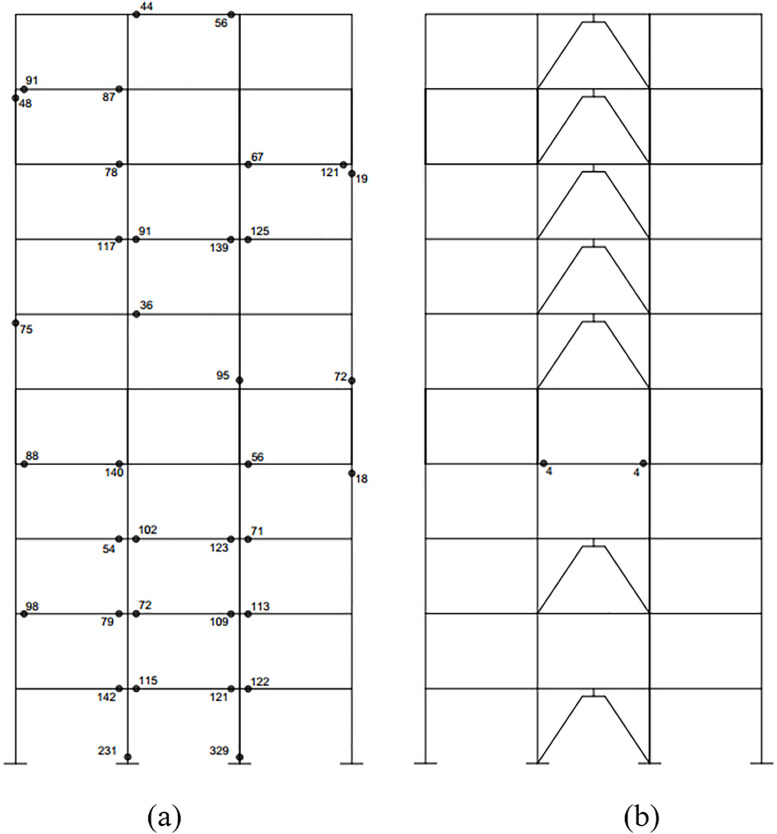
Comparison of hysteretic energy dissipation at each plastic hinge of the 10-story frame (a) without RFD (b) with optimal RFDs (unit: Kips.in).

The findings indicate that the hysteretic energy of the SMRF outfitted with the optimal RFDs is substantially reduced during the simulated seismic event. Consequently, this structural configuration can be safeguarded against significant structural damages. Fig 20 presents a comparison of the hysteretic energy dissipation at the plastic hinges of the structure fitted with 7 optimal dampers against that of the structure lacking RFDs. It is noted that a considerable proportion of the structural components in the configuration with the 7 optimal dampers remain within the elastic limits, thereby shielding the frame from severe damage.

**Fig 21:**
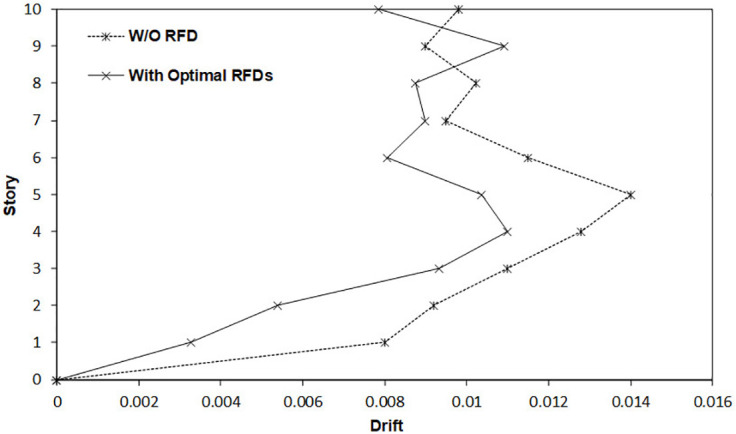
Comparative assessment of maximum structural drift with and without optimally designed RFDs.

Moreover, Fig 21 delineates the maximum inter-story drift ratio for the 10-story SMRF both without RFDs and with the 7 optimized RFDs. The figure clearly demonstrates that the integration of RFDs leads to a marked reduction in the inter-story drift ratio, underscoring their efficacy. However, in the upper stories, particularly story number 9, the inter-story drift ratio of the frame with RFDs exceeds that of the frame without RFDs. This variance is attributable to the fact that the optimization process is predominantly energy-focused, resulting in a compromise in drift reduction at elevated levels.

## 9. Conclusions

This research introduces a robust and comprehensive optimization strategy for the efficient design and strategic placement of rotational friction dampers (RFDs) within special moment-resisting frames (SMRFs), aiming to enhance their seismic performance. Leveraging a hybrid binary–real-coded particle swarm optimization (BRPSO) algorithm in conjunction with a seismic energy-based objective function, the study successfully identifies optimal damper configurations that significantly improve energy dissipation capacity while concurrently minimizing structural damage and inter-story drift. Through detailed sensitivity analyses, the study confirms that key RFD parameters—specifically the frictional moment (*M*_*f*_) and the length of the rigid beam (*h*_*a*_), have a profound impact on the dampers’ capacity to attenuate seismic responses. These parameters emerge as critical design variables in optimizing the seismic performance of SMRFs. Furthermore, by incorporating the effects of soil-structure interaction (SSI), the research highlights the necessity of accounting for foundation flexibility and ground conditions, which substantially influence overall structural response and energy dissipation behavior during seismic events. Importantly, the findings reveal that simply increasing the number of dampers does not linearly enhance seismic performance. Beyond an optimal threshold, additional dampers contribute minimally to further reductions in structural demand. This underscores the importance of precise, performance-based damper allocation, as opposed to uniform or excessive deployment, thereby supporting both economic efficiency and structural effectiveness. Comparative performance assessments demonstrate that the optimized SMRFs achieve marked reductions in both hysteretic energy demands and residual deformations, reflecting notable improvements in structural stability across various earthquake scenarios. These improvements affirm the efficacy of the proposed optimization framework as a practical and scalable solution for the development of high-performance seismic protection systems. Ultimately, the presented methodology offers valuable insights for the design of cost-effective, resilient structural systems in mid- to high-rise buildings situated in seismically active regions.
